# Supplementary material for: Pregnancy outcomes of immigrant women living in Korea: A population-based study
Source: PLoS One. 2022 Nov 29;17(11):e0278193. doi: 10.1371/journal.pone.0278193 (PMC9707740; doi:10.1371/journal.pone.0278193)
Supplement: Attachment — Submitted filename: PLOSONE2021.docx [file pone.0278193.s001.docx]

This article addresses an actual problem: the pregnancy outcomes among immigrant women in Korea. There are a number of issues with the methods and analysis that need to be clarified/addressed. Below are more specific comments by section:

**Abstract:**

Since you are calculating the OR, you should consider replacing “risk” by “odds”.

**Introduction**

I do not see what does the information about interracial marriages adds in the section.

The authors take a rather narrow view of data publication,

**Materials & Methods / Datasets and outcomes**

You are not describing from where you are taking the variable age from.

**Results section**

This section needs to be improved. The variables used for describing the study population are not exhaustive. If available, authors could also present information on employment status and educational level, previous c-section, Hypertension. Diabetes, for basic characteristics and potential risk factors. If you do not have information on these previous risk factors, you might want to mention it as a limitation.

Information regarding the % of missing data on each variable (you refer to this in the discussion section) could be useful

Table 1 includes information about outcomes that shouldn’t be described here. (GBD, preeclampsia. CS, induction etc)

When describing table 2 authors should refer as odds in state of risk. You might want to include information on low birth weigh and/or preterm birth since part of your background information includes those outcomes.

**Discussion section**

The first paragraph is confusing. Which are you main findings?

Line 179. You are including new information (birthweight) that wan not metion on the results section.

Try to stress what your data adds to the existing body of evidence
