# Supplementary material for: Pregnancy outcomes of immigrant women living in Korea: A population-based study
Source: PLoS One. 2022 Nov 29;17(11):e0278193. doi: 10.1371/journal.pone.0278193 (PMC9707740; doi:10.1371/journal.pone.0278193)
Supplement: Attachment — Submitted filename: response to reviewer plos one.docx [file pone.0278193.s002.docx]

Reviewer #1: This article addresses an actual problem: the pregnancy outcomes among immigrant women in Korea. There are a number of issues with the methods and analysis that need to be clarified/addressed. Below are more specific comments by section:
Abstract:
Since you are calculating the OR, you should consider replacing “risk” by “odds”.

Response: As you have pointed out, we wrote “odds” instead of “risk” as suggested.

Introduction
I do not see what does the information about interracial marriages adds in the section.
The authors take a rather narrow view of data publication,

Response: We fully agree with your suggestion. We corrected the introduction as follows (Line 65-75)

Preterm birth and low birth weight are serious adverse pregnancy outcomes. They are not only related to infant morbidity and mortality but also increase risk for chronic diseases such as cardiovascular diseases and metabolic syndrome during lifetime[1]. Several biological risk factors have been identified such as maternal age, body mass index and smoking and alcohol consumption. Further influences of racial and ethnic disparities have been documented. Although, many obstetric investigations across different countries have limited adjustment for ethnicity, compared to native women, immigrant women have an increased risk of preterm birth, low birth weight and cesarean section.[1,2, 4,5] It has been reported that immigrants in countries of resettlement have a greater risk of gestational diabetes mellitus (GDM) than women in receiving countries.[3] Thus, influxes of migrant women of childbearing age to receiving countries have made their perinatal health status a key priority for many governments.[5]

Materials & Methods / Datasets and outcomes
You are not describing from where you are taking the variable age from.

Response: We added “HIRA” in this sentence (Line 94-95) as following: “The HIRA database we received included unidentifiable codes that represented individuals, together with age, diagnosis, and a list of prescribed procedures.”

Results section
This section needs to be improved. The variables selected for describing the study population are not exhaustive. If available, authors could also present information on employment status and educational level, previous c-section, Hypertension. Diabetes, for basic characteristics and potential risk factors. If you do not have information on these previous risk factors, you might want to mention it as a limitation.

Response: We included limited information obtained from HIRA database and wrote limitation of certain risk factors (Line 201-205) in discussion. “Second, although the HIRA database comprises representative data of the whole country, it lacks information on uninsured medical claims, employment status, educational level as well as clinical information before the period we studied. In particular, migrant women do not know their past history in their previous country. ”

Information regarding the % of missing data on each variable (you refer to this in the discussion section) could be useful
Response: The strength of our study is that there is no missing data in HIRA database. We added this comment on page 13, line 206-207.

Table 1 includes information about outcomes that shouldn’t be described here. (GBD, preeclampsia. CS, induction etc)
Response: We reorganized table 1 into table 1 which includes basic characteristics and table 2 which includes pregnancy outcome.

When describing table 2 authors should refer as odds in state of risk. You might want to include information on low birth weigh and/or preterm birth since part of your background information includes those outcomes.
Response: We changed the risk to odds in Line 134-142.

Discussion section
The first paragraph is confusing. Which are you main findings?
Response: We clarified the first paragraph of discussion as follows.

“Immigrant women had a lower prevalence of preeclampsia and PPH and higher prevalence of GDM, CS and placenta previa compared to Korean women after adjustment for age, parity, and CCI.”

Line 179. You are including new information (birthweight) that was not mention on the results section.
Response: We deleted this irrelevant sentence. “In line with this, we previously reported that the birthweight was lower for the children of foreign women than for those of Korean women.”

Try to stress what your data adds to the existing body of evidence
Response: Our study with large population data demonstrated different pregnancy outcomes of immigrant women. Increased risk of GDM, CS and placenta previa were showed after adjusting maternal age, parity and CCI. This result will help clinicians to consider different pregnancy outcomes when they meet immigrant women. Eventually, predicting and preparing for adverse pregnancy outcome in advance will contribute to improving pregnancy outcomes.

Reviewer #2: The authors made a great presentation of the study findings - clearly linking the problem, aims, research questions, methodology, analysis results/findings, discussions, conclusions, and recommendations. Also, study clearly demonstrated associating factors with pregnancy outcomes in both groups. However, authors could provide more details in explanation to help the audience understand how caesarean section rate affects pregnancy outcome among immigrant women and state which specific cofounding factors affected (increased) caesarean section among immigrant women [line 176 & 177].
Response: We added following statement in Line 176-179. “In this study, the cesarean section rate was lower in immigrant women than in Korean women, but after adjustment for age, parity and CCI, the risk for cesarean section was higher in immigrant women. Immigrant women were younger than Korean women therefore age might be a factor which influences the lower rate of CS before adjustment.”

Over all, I think this is a technically sound and well documented study with details clearly stated findings and recommendations. Authors should state in the conclusion who (government, health workers, policy makers,…) is responsible for implementing effective pregnancy and postpartum management change/intervention for immigrant women [line 206 & 207] to enhance social change.

Response: We added following statement in the last paragraph of this manuscript(Line 207-209). “These characteristics should be reflected in Korean medical system to enhance social changes and effective pregnancy and postpartum management by healthcare providers will be necessary for immigrant women.”
