# Supplementary material for: Pregnancy outcomes of immigrant women living in Korea: A population-based study
Source: PLoS One. 2022 Nov 29;17(11):e0278193. doi: 10.1371/journal.pone.0278193 (PMC9707740; doi:10.1371/journal.pone.0278193)
Supplement: Attachment — Submitted filename: koren and inmigrants_2nd.docx [file pone.0278193.s003.docx]

The paper needs language editing. Consider a language revision.

When explaining The Charlson Comorbidity Index it would be useful to explain if the higher the score the more comorbid conditions are present (line 110)

From line 135 to 140 you described table 1, but in the middle of the paragraph there is information related to table 2 (The cesarean section (CS) rate was lower in foreign women than Korean women. The incidence of placental abruption, placental previa, PPH, PH, and UAE

were lower in immigrant women than Korean women.) that information was repeated in line 150.

Table 2. you included: Values are expressed as mean (SD) or %. But there are not values expressed as mean and SD

Table 3. should be named: Odds ratio and 95% confidence intervals of adverse pregnancy out 155 comes of immigrant women instead of Odds ratio and 95% confidence intervals of the risk of adverse pregnancy out 155 comes of immigrant women

This sentence is confusing:

In particular, migrant women do not know their past history in their previous country

Which is their past history they don’t know?

In table 1 it says “Primparity” check spelling

Line 187: it says risks, it should say odds

190. I still see the sentences: In line with this, we previously reported that the birthweight was lower for the children of foreign women than for those of Korean women.[11]

In this sense: I asked you before, Try to stress what your data adds to the existing body of evidence

Your Response was: “Our study with large population data demonstrated different pregnancy

outcomes of immigrant women. Increased risk of GDM, CS and placenta previa were

showed after adjusting maternal age, parity and CCI. This result will help clinicians to

consider different pregnancy outcomes when they meet immigrant women. Eventually,

predicting and preparing for adverse pregnancy outcome in advance will contribute to

improving pregnancy outcomes.”

That information was not included in the discussion section. Besides, there was not an increased risk of placenta previa (the confidence interval starts in1)

I think you should do emphasis on the results presented in table 3.: After adjusting by age, parity and CCI, which are the variables you are expected to adjust by, (considering there were differences between the 2 groups in table 1). Findings are that: to be immigrant increase the risk of having GDM, and CS. But protects against having preeclampsia, and induction.
