# Supplementary material for: Pregnancy outcomes of immigrant women living in Korea: A population-based study
Source: PLoS One. 2022 Nov 29;17(11):e0278193. doi: 10.1371/journal.pone.0278193 (PMC9707740; doi:10.1371/journal.pone.0278193)
Supplement: Attachment — Submitted filename: Response to reviewers after 2nd.docx [file pone.0278193.s004.docx]

Response to reviewers

Reviewer #1: The paper needs language editing. Consider a language revision.

: We finished language editing as suggested.

When explaining The Charlson Comorbidity Index it would be useful to explain if the higher the score the more comorbid conditions are present (line 110)

: We added this statement in line 112 “that the higher the score the more comorbid conditions are present’

From line 135 to 140 you described table 1, but in the middle of the paragraph there is information related to table 2 (The cesarean section (CS) rate was lower in foreign women than Korean women. The incidence of placental abruption, placental previa, PPH, PH, and UAE

were lower in immigrant women than Korean women.) that information was repeated in line 150.

:We deleted this sentence in line 130.

Table 2. you included: Values are expressed as mean (SD) or %. But there are not values expressed as mean and SD

: We wrote “values are expressed as number, %.”

Table 3. should be named: Odds ratio and 95% confidence intervals of adverse pregnancy out 155 comes of immigrant women instead of Odds ratio and 95% confidence intervals of the risk of adverse pregnancy out 155 comes of immigrant women

: We corrected as suggested.

This sentence is confusing:

In particular, migrant women do not know their past history in their previous country

Which is their past history they don’t know?

: We corrected this sentence as follows; In particular it was difficult to obtain previous medical histories of migrant women in their previous country.

In table 1 it says “Primparity” check spelling

: We corrected as suggested

Line 187: it says risks, it should say odds 190.

: We corrected as suggested

I still see the sentences: In line with this, we previously reported that the birthweight was lower for the children of foreign women than for those of Korean women.[11]

: We deleted this sentence as suggested.

In this sense: I asked you before, Try to stress what your data adds to the existing body of evidence

Your Response was: “Our study with large population data demonstrated different pregnancy

outcomes of immigrant women. Increased risk of GDM, CS and placenta previa were

showed after adjusting maternal age, parity and CCI. This result will help clinicians to

consider different pregnancy outcomes when they meet immigrant women. Eventually,

predicting and preparing for adverse pregnancy outcome in advance will contribute to

improving pregnancy outcomes.”

That information was not included in the discussion section. Besides, there was not an increased risk of placenta previa (the confidence interval starts in1)

: We added this sentences in discussion section. The risk of placenta previa was corrected in abstract, results and discussion.

I think you should do emphasis on the results presented in table 3.: After adjusting by age, parity and CCI, which are the variables you are expected to adjust by, (considering there were differences between the 2 groups in table 1). Findings are that: to be immigrant increase the risk of having GDM, and CS. But protects against having preeclampsia, and induction.

: We added following statement in the first paragraph of discussion (Line 166-168)

Immigrant women were protected against having preeclampsia and PPH and higher risk of GDM and CS compared to Korean women after adjustment for age, parity and CCI.
