# Supplementary material for: Pregnancy outcomes of immigrant women living in Korea: A population-based study
Source: PLoS One. 2022 Nov 29;17(11):e0278193. doi: 10.1371/journal.pone.0278193 (PMC9707740; doi:10.1371/journal.pone.0278193)
Supplement: Attachment — Submitted filename: Response to reviewers.docx [file pone.0278193.s005.docx]

Response to reviewers

Reviewer #3: The authors have done well in providing evidence on pregnancy outcomes among immigrants in Korea. Authors could consider the following issues to strengthen the paper.

Introduction

Authors provide a very brief introduction to the topic of interest and seem to miss out important detail. First, about half of the first paragraph focuses on preterm birth and low birth weight and their predictors when the study itself (including analysis) rarely considers these outcomes. Second, the detail on interracial marriage in Korea is great to provide information on the immigration status in Korea. However, the argument is too one-sided and does not feed into the issues raised in the subsequent sentences. I think a reviewer in the earlier revisions of this work raised this issue. Authors could strengthen their introduction by considering the following:

1. What pregnancy outcomes are highly prevalent in Korea?

🡪 In South Korea, the incidence of GDM, hypertensive disorder in pregnancy, and preterm birth and the rate of cesarean section have been steadily increasing. GDM accounts for 15.8% in 2017 to 18.01% in 2021, preeclampsia has doubled 2.75% in 2017 to 5.34% in 2021, preterm birth rate was 5.8% in 2010 and 8.5% in 2020

2. Are there studies that report on these outcomes by immigration status in Korea? Kindly report on this in addition to what has been done in other countries.

3. Justify why immigrants in Korea should be focused.

🡪 We appreciate above suggestions mainly focusing on why we need to study immigrant pregnant women and their association with adverse pregnancy outcomes. Therefore we rewrote and organized introduction.

Methods

1. Authors should pro vide justification for the duration 2007 - 2016.

🡪 At the time of planning the study, period between 2007 and 2016 could be accessed and analyzed. In the future, it is also necessary to use this data to see long-term changes.

2. Can authors clearly define their outcome variables? For instance a number of variables are mentioned and this includes parity and multiple pregnancies which i presume are not the main outcome variables of interest they seek to look at.

🡪 We defined variables we looked at as suggested in methods

🡪 Parity and the rate of cesarean section, induction, vacuum delivery, uterine arterial embolization (UAE), and peripartum hysterectomy (PH) were also identified using the presence of a Korea Medical Insurance electronic data interchange (EDI) code.

3. Regarding section titled "covariates", authors seem to focus mainly on ICC and how it is created from pre-pregnancy factors but there is no information on the specific factors that were combined to create this index. Neither is there information on how this index is created. Is this something that exist in the database or it is an index authors created themselves. Authors should kindly provide more detail regarding this.

🡪 CCI was used in this manuscript to represent comorbid status of patients before pregnancy as a simple and widely used method to identify underlying diseases. We added more detail and reference on CCI in the methods.

🡪The CCI has been known to be a useful and most widely used tool to measure comorbid disease status including cardiovascular diseases, diabetes, malignancies and autoimmune diseases or casemix in health care databases that the higher the score the more comorbid conditions are present. Acute myocardial infarction, Congestive heart failure, Peripheral vascular disease, Cerebral vascular accident, Dementia, Pulmonary disease, Connective tissue disorder, Peptic ulcer, Liver disease, Diabetes, Diabetes complications, Paraplegia, Renal disease, Cancer, Metastatic cancer, Severe liver disease, HIV were analyzed in this study.

4. Since the data for the study covers the period between 2007 and 2016, there is the possibility of multiple pregnancies per woman. How do authors account for this?

🡪 Multiple pregnancies were included in this data set but we did not put as a variable. We also deleted “multiple pregnancy” in method section.

5. Line 118: can authors specify the exact group they are referring to?

🡪 We added following sentence to define group

🡪 (Line 130-131) The Student’s t-test was used to compare continuous variables between Korean and immigrant pregnant women

6. Authors also do not mention the final sample size they used.

🡪 Final sample size was 4,439,778.

7. I think authors should dedicate a section for ethical consideration to outline all forms of ethics that apply to their work; data availability and how it was accessed.

🡪 We added following statement in method.

🡪 **Ethical consideration**

This study was approved by the Institutional Review Committees of Korea University Guro Hospital (KUGH17273). The informed consent was waived by this IRB because the HIRA database does not include individual identities and because of retrospective nature of this study. This study comprises of third-party data therefore authors cannot share data nor legally distribute.

Results

1. lines 131-133: results is not in Table 1. Authors should check this and interpret the other variables in table one in place of this.

🡪 We corrected result of table 1 in manuscript.

🡪 Table 1 shows the basic characteristics of the study population according to maternal ethnicity. Foreign women were younger and more primiparous than Korean women and tended to have lower CCI score than Korean women.

Discussion

I find the results to be well discussed and the limitations well laid out.

🡪We appreciate this comment.

Reviewer #4: I appreciate the opportunity to review your manuscript. Please find below some important areas in which the manuscript can be clarified or improved from the statistical perspective.

1) The methods section of the manuscript can be better organized by using sub headings e.g. study design and data source, study outcomes, other measures, etc. Please follow the CONSORT guidelines or any other required by the journal.

🡪 We used following headings in methods.

Study desing-data characteristics-Ethical consideration-Dataset and outcomes-Covariates-Statistical analysis

2) Primary and secondary outcomes must be clearly identified and distinguished in the abstract, methods section, statistical methods, results, and discussion.

🡪 We used ‘main outcome’ and added following sentence in the abstract, method, results and discussion.

🡪 Main outcome of this study were adverse pregnancy outcomes including GDM, preeclampsia, cesarean section, induction of labor, vacuum delivery, placenta previa, placenta abruption, PPH, peripartum hysterectomy and UAE. Age, parity and CCI were adjusted for multiple logistic regression analysis.

3) Please mention all the covariates that were examined in the covariates section of the manuscript.

🡪 CCI used in this study included following diseases.

🡪 Acute myocardial infarction, Congestive heart failure, Peripheral vascular disease, Cerebral vascular accident, Dementia, Pulmonary disease, Connective tissue disorder, Peptic ulcer, Liver disease, Diabetes, Diabetes complications, Paraplegia, Renal disease, Cancer, Metastatic cancer, Severe liver disease, HIV were analyzed in this study.

4) In the statistical analysis section, please include that model was run stepwise and reason behind running 3 separate models.

🡪We added ‘the model was run stepwise’ in method. The running 3 separate models was conducted to identify the variables that affect the most.

5) In all the tables in which statistical tests were conducted, the footnotes need to specify which test was used to derive each reported P-value.

🡪 We added all the statistical tests used in the footnotes of each tables.

6) For age please clarify in the tables if it is mean age or median.

🡪 For age, the results indicate mean±standard deviation.

7) Authors have multiple outcome variables but use 0.05 as significance criterion throughout. Please use Bonferroni correction or other method to adjust for multiple testing. Otherwise, the results will have an elevated chance of being false positives.

🡪 We appreciate thorough statistical suggestions. In this study, multiple outcome variables were analyzed. Although each variable may be related to each other, each variable is an important subject identified in obstetrics. Therefore, age, parity, and CCI were adjusted in regression model.

8) Was there any missing data in your study? How was it handled?

🡪This study is based on the claim data and since the diagnosis was confirmed using the icd-10 code, there is no missing data among the mothers charged for delivery. We added this issue in limitation of discussion.
