# Supplementary material for: Pregnancy outcomes of immigrant women living in Korea: A population-based study
Source: PLoS One. 2022 Nov 29;17(11):e0278193. doi: 10.1371/journal.pone.0278193 (PMC9707740; doi:10.1371/journal.pone.0278193)
Supplement: Attachment — Submitted filename: Third review_immigrant Preg outcomes.docx [file pone.0278193.s006.docx]

In the introduction section from line 88 to line 90 there are empty lines.

Line 147 is should be resultS

**Table 2.**  should be named**: “pregnancy outcomes of the study population**” without “The”

Table 3. should be named: Odds ratio and 95% confidence intervals of adverse pregnancy outcomes of immigrant women. Check out please you included an extra “155”. It was a cut & paste mistake for sure.

Line 198. Table 3 does not “represents”, I guess you wanted to say “presents”

Also in line 198, I would add the word “unadjusted” in this sentence: “The odds of GDM, preeclampsia, CS, placental abruption, placental previa, PPH, PH, and UAE was lower in foreign women than Korean women.”

In line 215 and 216 why you did not mention the protection against induction?

“Immigrant women were protected against having preeclampsia and PPH and

216 higher risk of GDM, and CS compared to Korean women after adjustment for age, parity, and CCI.”

Line 261 it should be outcomeS

There are still some spelling mistakes. I suggest you perform a language edition to your manuscript.
